# *N*,*N*,*N*′,*N*′-Tetra­isobutyl­pyridine-2,6-dicarboxamide

**DOI:** 10.1107/S1600536811040608

**Published:** 2011-10-08

**Authors:** Michaela Pojarová, Michal Dušek, Emanuel Makrlík, Vasily A. Babain, Petr Vaňura

**Affiliations:** a Institute of Physics, AS CR, v.v.i., Na Slovance 2, 182 21 Praha 8, Czech Republic; b Faculty of Environmental Sciences, Czech University of Life Sciences, Prague, Kamýcká 129, 165 21 Prague 6, Czech Republic; c Khlopin Radium Institute, Research and Production Association, 2nd Murinskiy Prospect b. 28, 194021 St Petersburg, Russian Federation; d Department of Analytical Chemistry, Institute of Chemical Technology, Prague, Technická 5, 166 28 Prague 6, Czech Republic

## Abstract

In the title compound, C_23_H_39_N_3_O_2_, the amide O atoms are displaced by 1.020 (1) and 1.211 (1) Å from the mean plane of the central pyridine ring. In the crystal, mol­ecules are connected by weak C—H⋯O hydrogen bonds between methyl­ene groups in the isobutyl substituents and the amide O atoms.

## Related literature

The title compound has been investigated for its extractive properties towards trivalent metals in a synergistic mixture with chlorinated cobalt dicarbollide. For further information, see: Alyapyshev *et al.* (2004 ▶, 2006 ▶); Romanovskiy *et al.* (2006 ▶); Babain *et al.* (2007 ▶); Makrlík *et al.* (2009 ▶, 2011 ▶). For further synthetic details, see: Nikitskaya *et al.* (1958 ▶); Shimada *et al.* (2004 ▶).
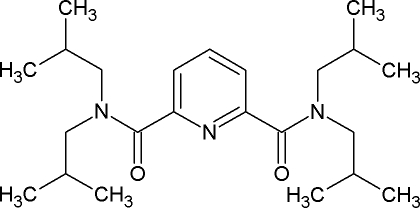


## Experimental

### 

#### Crystal data

C_23_H_39_N_3_O_2_
*M* *_r_* = 389.57
Monoclinic, 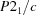

*a* = 10.5247 (2) Å
*b* = 17.7765 (3) Å
*c* = 12.8773 (2) Å
β = 96.877 (2)°
*V* = 2391.91 (7) Å^3^
*Z* = 4
Cu *K*α radiation
μ = 0.54 mm^−1^
*T* = 120 K
0.49 × 0.42 × 0.23 mm

#### Data collection

Agilent Xcalibur Atlas Gemini ultra diffractometer
Absorption correction: analytical (*CrysAlis PRO*; Agilent, 2011 ▶) *T*_min_ = 0.929, *T*_max_ = 0.962
31214 measured reflections
4272 independent reflections
3867 reflections with *I* > 2σ(*I*)
*R*_int_ = 0.039

#### Refinement

*R*[*F* ^2^ > 2σ(*F* ^2^)] = 0.040
*wR*(*F* ^2^) = 0.112
*S* = 1.05
4272 reflections
261 parameters
H-atom parameters constrained
Δρ_max_ = 0.20 e Å^−3^
Δρ_min_ = −0.14 e Å^−3^

### 

Data collection: *CrysAlis PRO* (Agilent, 2011 ▶); cell refinement: *CrysAlis PRO*; data reduction: *CrysAlis PRO*; program(s) used to solve structure: *SHELXS97* (Sheldrick, 2008 ▶); program(s) used to refine structure: *SHELXL97* (Sheldrick, 2008 ▶); molecular graphics: *Mercury* (Macrae *et al.*, 2006 ▶) and *DIAMOND* (Brandenburg & Putz, 2005 ▶); software used to prepare material for publication: *publCIF* (Westrip, 2010 ▶).

## Supplementary Material

Crystal structure: contains datablock(s) I, global. DOI: 10.1107/S1600536811040608/hb6423sup1.cif

Structure factors: contains datablock(s) I. DOI: 10.1107/S1600536811040608/hb6423Isup2.hkl

Supplementary material file. DOI: 10.1107/S1600536811040608/hb6423Isup3.cml

Additional supplementary materials:  crystallographic information; 3D view; checkCIF report

## Figures and Tables

**Table 1:** Hydrogen-bond geometry (Å, °)

*D*—H⋯*A*	*D*—H	H⋯*A*	*D*⋯*A*	*D*—H⋯*A*
C7—H7*A*⋯O2^i^	0.97	2.57	3.5226 (15)	166
C11—H11*A*⋯O1^ii^	0.97	2.55	3.4790 (15)	160

Symmetry codes: (i) 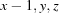; (ii) 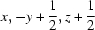.

**Fig. 1.**
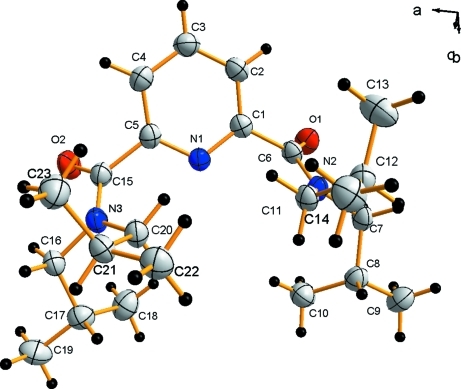
View of the N,N,N',N'-tetraisobutyl-2,6-dipicolinamide, together with atom-labelling scheme. Displacement ellipsoids are shown at the 50% probability level.

**Fig. 2.**
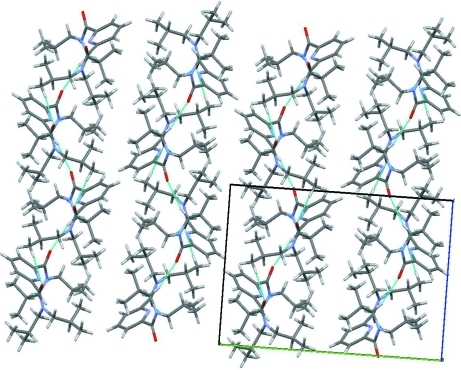
Projection along the b axis with highlighted hydrogen bonds between the molecules in the bands in direction of c axis.

## supplementary crystallographic information

### Comment

The title compound, (I), shown in Figure 1 and Scheme 1, has been investigated in a synergistic mixtures with the dicarbollylcobaltate anion and its halogen derivatives for significant extraction properties towards trivalent metal cations (Alyapyshev *et al.*,2004). It consists of pyridine ring with a di-isobutylamide groups in position 2 and 6. This molecule lacks of crystallographic symmetry and the asymmetric unit contains one molecule. While at first impression, the amide groups seem to be related by a mirror plane, closer look reveales their differences. The carbon atoms of carbonyl groups do not lay in a plane of the pyridine ring and they differ in the distance to this plane (0.062 Å for C6 and 0.234 Å for C15). The molecules form bands along the *c* axis (Fig. 2) *via* system of hydrogen bonds (Table 1).

### Experimental

The title compound was synthesized as described in Shimada *et al.* (2004), and Nikitskaya *et al.* (1958).  Colourless prisms were prepared by slow evaporation from an acetonitrile solution.

### Refinement

The hydrogen atoms were localized from the difference Fourier map. Despite of that, all hydrogen atoms connected to C were constrained to ideal positions. The isotropic temperature parameters of hydrogen atoms were calculated as 1.2**U*_eq_ of the parent atom.

### Crystal data

**Table:**

C_23_H_39_N_3_O_2_	*F*(000) = 856
*M**_r_* = 389.57	*D*_x_ = 1.082 Mg m^−^^3^
Monoclinic, *P*2_1_/*c*	Cu *K*α radiation, λ = 1.5418 Å
Hall symbol: -P 2ybc	Cell parameters from 15197 reflections
*a* = 10.5247 (2) Å	θ = 3.5–67.0°
*b* = 17.7765 (3) Å	µ = 0.54 mm^−^^1^
*c* = 12.8773 (2) Å	*T* = 120 K
β = 96.877 (2)°	Prism, colourless
*V* = 2391.91 (7) Å^3^	0.49 × 0.42 × 0.23 mm
*Z* = 4	

### Data collection

**Table:**

Agilent Xcalibur Atlas Gemini ultra diffractometer	4272 independent reflections
Radiation source: Enhance Ultra (Cu) X-ray Source	3867 reflections with *I* > 2σ(*I*)
mirror	*R*_int_ = 0.039
Detector resolution: 10.3784 pixels mm^-1^	θ_max_ = 67.1°, θ_min_ = 4.2°
Rotation method data acquisition using ω scans	*h* = −12→12
Absorption correction: analytical (*CrysAlis PRO*; Agilent, 2011)	*k* = −20→21
*T*_min_ = 0.929, *T*_max_ = 0.962	*l* = −15→15
31214 measured reflections	

### Refinement

**Table:**

Refinement on *F*^2^	Primary atom site location: structure-invariant direct methods
Least-squares matrix: full	Secondary atom site location: difference Fourier map
*R*[*F*^2^ > 2σ(*F*^2^)] = 0.040	Hydrogen site location: inferred from neighbouring sites
*wR*(*F*^2^) = 0.112	H-atom parameters constrained
*S* = 1.05	*w* = 1/[σ^2^(*F*_o_^2^) + (0.0596*P*)^2^ + 0.5367*P*] where *P* = (*F*_o_^2^ + 2*F*_c_^2^)/3
4272 reflections	(Δ/σ)_max_ < 0.001
261 parameters	Δρ_max_ = 0.20 e Å^−^^3^
0 restraints	Δρ_min_ = −0.14 e Å^−^^3^

### Special details

**Table:**

Geometry. All e.s.d.'s (except the e.s.d. in the dihedral angle between two l.s. planes) are estimated using the full covariance matrix. The cell e.s.d.'s are taken into account individually in the estimation of e.s.d.'s in distances, angles and torsion angles; correlations between e.s.d.'s in cell parameters are only used when they are defined by crystal symmetry. An approximate (isotropic) treatment of cell e.s.d.'s is used for estimating e.s.d.'s involving l.s. planes.
Refinement. Refinement of *F*^2^ against ALL reflections. The weighted *R*-factor *wR* and goodness of fit *S* are based on *F*^2^, conventional *R*-factors *R* are based on *F*, with *F* set to zero for negative *F*^2^. The threshold expression of *F*^2^ > σ(*F*^2^) is used only for calculating *R*-factors(gt) *etc*. and is not relevant to the choice of reflections for refinement. *R*-factors based on *F*^2^ are statistically about twice as large as those based on *F*, and *R*- factors based on ALL data will be even larger. The hydrogen atoms were localized from the difference Fourier map. Despite of that, all hydrogen atoms connected to C were constrained to ideal positions. The isotropic temperature parameters of hydrogen atoms were calculated as 1.2**U*_eq_ of the parent atom.

### Fractional atomic coordinates and isotropic or equivalent isotropic displacement parameters (Å^2^)

**Table:**

	*x*	*y*	*z*	*U*_iso_*/*U*_eq_	
C1	0.59347 (11)	0.14933 (7)	0.60811 (8)	0.0311 (3)	
C2	0.60691 (12)	0.08382 (7)	0.55206 (9)	0.0371 (3)	
H2	0.5408	0.0668	0.5033	0.044*	
C3	0.72048 (12)	0.04424 (7)	0.57014 (10)	0.0402 (3)	
H3	0.7308	−0.0011	0.5361	0.048*	
C4	0.81870 (12)	0.07328 (7)	0.63991 (10)	0.0371 (3)	
H4	0.8965	0.0482	0.6529	0.045*	
C5	0.79847 (11)	0.14055 (7)	0.68995 (9)	0.0319 (3)	
C6	0.47213 (11)	0.19491 (7)	0.58800 (9)	0.0329 (3)	
N2	0.40863 (9)	0.21191 (6)	0.66956 (7)	0.0332 (2)	
C7	0.30023 (11)	0.26406 (7)	0.65316 (9)	0.0363 (3)	
H7A	0.2221	0.2366	0.6604	0.044*	
H7B	0.2939	0.2832	0.5822	0.044*	
C8	0.31001 (12)	0.33034 (8)	0.72858 (10)	0.0396 (3)	
H8	0.3019	0.3115	0.7990	0.048*	
C9	0.19691 (14)	0.38209 (9)	0.69475 (12)	0.0509 (4)	
H9A	0.2051	0.4023	0.6267	0.061*	
H9B	0.1958	0.4225	0.7441	0.061*	
H9C	0.1187	0.3540	0.6922	0.061*	
C10	0.43658 (14)	0.37205 (8)	0.73123 (12)	0.0492 (3)	
H10A	0.5058	0.3383	0.7532	0.059*	
H10B	0.4381	0.4133	0.7795	0.059*	
H10C	0.4457	0.3909	0.6627	0.059*	
C11	0.43174 (11)	0.17506 (7)	0.77252 (9)	0.0337 (3)	
H11A	0.4472	0.2134	0.8261	0.040*	
H11B	0.5083	0.1444	0.7748	0.040*	
C12	0.32074 (12)	0.12556 (7)	0.79723 (10)	0.0383 (3)	
H12	0.2446	0.1573	0.7971	0.046*	
C13	0.29120 (17)	0.06398 (9)	0.71727 (12)	0.0577 (4)	
H13A	0.3641	0.0315	0.7176	0.069*	
H13B	0.2715	0.0859	0.6491	0.069*	
H13C	0.2191	0.0353	0.7342	0.069*	
C14	0.35334 (15)	0.09285 (8)	0.90645 (11)	0.0492 (3)	
H14A	0.2806	0.0662	0.9261	0.059*	
H14B	0.3756	0.1328	0.9553	0.059*	
H14C	0.4244	0.0589	0.9068	0.059*	
C15	0.90910 (11)	0.17978 (7)	0.75374 (10)	0.0352 (3)	
N3	0.89638 (9)	0.20256 (6)	0.85171 (8)	0.0351 (2)	
C16	0.99475 (11)	0.25225 (7)	0.90474 (10)	0.0375 (3)	
H16A	1.0630	0.2581	0.8610	0.045*	
H16B	1.0309	0.2288	0.9696	0.045*	
C17	0.94382 (13)	0.32978 (7)	0.92891 (10)	0.0407 (3)	
H17	0.8868	0.3241	0.9833	0.049*	
C18	0.86814 (15)	0.36539 (9)	0.83386 (12)	0.0528 (4)	
H18A	0.9224	0.3715	0.7796	0.063*	
H18B	0.8369	0.4137	0.8526	0.063*	
H18C	0.7972	0.3336	0.8092	0.063*	
C19	1.05650 (15)	0.37855 (8)	0.97250 (11)	0.0505 (4)	
H19A	1.1168	0.3817	0.9223	0.061*	
H19B	1.0973	0.3567	1.0361	0.061*	
H19C	1.0265	0.4281	0.9867	0.061*	
C20	0.78661 (11)	0.18394 (7)	0.90704 (9)	0.0357 (3)	
H20A	0.7305	0.1502	0.8638	0.043*	
H20B	0.7390	0.2296	0.9166	0.043*	
C21	0.82297 (11)	0.14717 (7)	1.01368 (9)	0.0355 (3)	
H21	0.8725	0.1832	1.0598	0.043*	
C22	0.69990 (13)	0.12864 (8)	1.05952 (10)	0.0417 (3)	
H22A	0.6509	0.1738	1.0644	0.050*	
H22B	0.7204	0.1073	1.1280	0.050*	
H22C	0.6508	0.0932	1.0151	0.050*	
C23	0.90338 (13)	0.07718 (8)	1.00455 (12)	0.0479 (3)	
H23A	0.8561	0.0417	0.9589	0.057*	
H23B	0.9241	0.0550	1.0725	0.057*	
H23C	0.9808	0.0905	0.9765	0.057*	
N1	0.68718 (9)	0.17810 (6)	0.67644 (7)	0.0313 (2)	
O1	0.43906 (9)	0.21594 (6)	0.49754 (6)	0.0446 (2)	
O2	1.00544 (8)	0.19100 (6)	0.71057 (8)	0.0493 (3)	

### Atomic displacement parameters (Å^2^)

**Table:**

	*U*^11^	*U*^22^	*U*^33^	*U*^12^	*U*^13^	*U*^23^
C1	0.0313 (6)	0.0360 (6)	0.0262 (5)	−0.0027 (5)	0.0038 (4)	0.0022 (4)
C2	0.0380 (6)	0.0393 (6)	0.0333 (6)	−0.0044 (5)	0.0017 (5)	−0.0033 (5)
C3	0.0435 (7)	0.0359 (6)	0.0420 (7)	−0.0001 (5)	0.0077 (5)	−0.0060 (5)
C4	0.0333 (6)	0.0390 (6)	0.0398 (6)	0.0034 (5)	0.0074 (5)	0.0010 (5)
C5	0.0288 (6)	0.0388 (6)	0.0286 (6)	−0.0008 (5)	0.0061 (4)	0.0018 (5)
C6	0.0320 (6)	0.0379 (6)	0.0277 (6)	−0.0019 (5)	−0.0008 (5)	−0.0007 (5)
N2	0.0284 (5)	0.0426 (6)	0.0275 (5)	0.0028 (4)	−0.0008 (4)	0.0013 (4)
C7	0.0281 (6)	0.0455 (7)	0.0340 (6)	0.0033 (5)	−0.0010 (5)	0.0020 (5)
C8	0.0372 (7)	0.0482 (7)	0.0340 (6)	0.0029 (5)	0.0067 (5)	0.0000 (5)
C9	0.0511 (8)	0.0551 (8)	0.0475 (8)	0.0133 (7)	0.0104 (6)	−0.0011 (6)
C10	0.0484 (8)	0.0493 (8)	0.0498 (8)	−0.0065 (6)	0.0057 (6)	−0.0062 (6)
C11	0.0301 (6)	0.0429 (7)	0.0273 (6)	0.0001 (5)	0.0002 (5)	0.0012 (5)
C12	0.0304 (6)	0.0401 (7)	0.0440 (7)	−0.0004 (5)	0.0035 (5)	0.0019 (5)
C13	0.0676 (10)	0.0462 (8)	0.0541 (9)	−0.0115 (7)	−0.0138 (7)	0.0009 (7)
C14	0.0543 (8)	0.0517 (8)	0.0430 (7)	−0.0097 (7)	0.0122 (6)	0.0033 (6)
C15	0.0274 (6)	0.0416 (7)	0.0367 (6)	0.0023 (5)	0.0038 (5)	−0.0011 (5)
N3	0.0262 (5)	0.0459 (6)	0.0329 (5)	−0.0011 (4)	0.0019 (4)	−0.0031 (4)
C16	0.0293 (6)	0.0433 (7)	0.0384 (6)	−0.0012 (5)	−0.0020 (5)	−0.0018 (5)
C17	0.0454 (7)	0.0428 (7)	0.0346 (6)	0.0012 (6)	0.0076 (5)	0.0022 (5)
C18	0.0537 (8)	0.0545 (8)	0.0510 (8)	0.0108 (7)	0.0092 (7)	0.0135 (6)
C19	0.0655 (9)	0.0442 (7)	0.0423 (7)	−0.0099 (7)	0.0081 (7)	−0.0019 (6)
C20	0.0281 (6)	0.0470 (7)	0.0318 (6)	0.0005 (5)	0.0022 (5)	0.0002 (5)
C21	0.0345 (6)	0.0388 (6)	0.0314 (6)	−0.0003 (5)	−0.0027 (5)	−0.0028 (5)
C22	0.0443 (7)	0.0485 (7)	0.0323 (6)	0.0010 (6)	0.0040 (5)	0.0024 (5)
C23	0.0421 (7)	0.0436 (7)	0.0568 (8)	0.0036 (6)	0.0010 (6)	0.0029 (6)
N1	0.0294 (5)	0.0378 (5)	0.0268 (5)	−0.0005 (4)	0.0036 (4)	0.0000 (4)
O1	0.0461 (5)	0.0595 (6)	0.0272 (4)	0.0104 (4)	0.0001 (4)	0.0047 (4)
O2	0.0304 (5)	0.0735 (7)	0.0459 (5)	−0.0086 (4)	0.0117 (4)	−0.0119 (5)

### Geometric parameters (Å, °)

**Table:**

C1—N1	1.3419 (15)	C13—H13B	0.9600
C1—C2	1.3862 (17)	C13—H13C	0.9600
C1—C6	1.5084 (16)	C14—H14A	0.9600
C2—C3	1.3825 (18)	C14—H14B	0.9600
C2—H2	0.9300	C14—H14C	0.9600
C3—C4	1.3856 (18)	C15—O2	1.2295 (15)
C3—H3	0.9300	C15—N3	1.3469 (16)
C4—C5	1.3868 (18)	N3—C20	1.4660 (15)
C4—H4	0.9300	N3—C16	1.4660 (16)
C5—N1	1.3412 (15)	C16—C17	1.5242 (18)
C5—C15	1.5125 (17)	C16—H16A	0.9700
C6—O1	1.2332 (14)	C16—H16B	0.9700
C6—N2	1.3450 (16)	C17—C18	1.5165 (19)
N2—C7	1.4655 (15)	C17—C19	1.521 (2)
N2—C11	1.4726 (15)	C17—H17	0.9800
C7—C8	1.5225 (18)	C18—H18A	0.9600
C7—H7A	0.9700	C18—H18B	0.9600
C7—H7B	0.9700	C18—H18C	0.9600
C8—C10	1.5214 (19)	C19—H19A	0.9600
C8—C9	1.5261 (19)	C19—H19B	0.9600
C8—H8	0.9800	C19—H19C	0.9600
C9—H9A	0.9600	C20—C21	1.5276 (17)
C9—H9B	0.9600	C20—H20A	0.9700
C9—H9C	0.9600	C20—H20B	0.9700
C10—H10A	0.9600	C21—C23	1.5172 (18)
C10—H10B	0.9600	C21—C22	1.5225 (18)
C10—H10C	0.9600	C21—H21	0.9800
C11—C12	1.5262 (17)	C22—H22A	0.9600
C11—H11A	0.9700	C22—H22B	0.9600
C11—H11B	0.9700	C22—H22C	0.9600
C12—C13	1.510 (2)	C23—H23A	0.9600
C12—C14	1.5226 (19)	C23—H23B	0.9600
C12—H12	0.9800	C23—H23C	0.9600
C13—H13A	0.9600		
			
N1—C1—C2	123.28 (11)	H13B—C13—H13C	109.5
N1—C1—C6	116.65 (10)	C12—C14—H14A	109.5
C2—C1—C6	119.93 (10)	C12—C14—H14B	109.5
C3—C2—C1	118.68 (11)	H14A—C14—H14B	109.5
C3—C2—H2	120.7	C12—C14—H14C	109.5
C1—C2—H2	120.7	H14A—C14—H14C	109.5
C2—C3—C4	118.85 (12)	H14B—C14—H14C	109.5
C2—C3—H3	120.6	O2—C15—N3	123.68 (11)
C4—C3—H3	120.6	O2—C15—C5	116.89 (11)
C3—C4—C5	118.58 (11)	N3—C15—C5	119.40 (10)
C3—C4—H4	120.7	C15—N3—C20	124.05 (10)
C5—C4—H4	120.7	C15—N3—C16	118.27 (10)
N1—C5—C4	123.31 (11)	C20—N3—C16	117.60 (10)
N1—C5—C15	116.39 (10)	N3—C16—C17	113.20 (10)
C4—C5—C15	119.92 (10)	N3—C16—H16A	108.9
O1—C6—N2	124.00 (11)	C17—C16—H16A	108.9
O1—C6—C1	117.54 (10)	N3—C16—H16B	108.9
N2—C6—C1	118.44 (10)	C17—C16—H16B	108.9
C6—N2—C7	118.72 (10)	H16A—C16—H16B	107.8
C6—N2—C11	124.00 (10)	C18—C17—C19	111.77 (12)
C7—N2—C11	116.93 (9)	C18—C17—C16	112.09 (11)
N2—C7—C8	113.93 (10)	C19—C17—C16	108.24 (11)
N2—C7—H7A	108.8	C18—C17—H17	108.2
C8—C7—H7A	108.8	C19—C17—H17	108.2
N2—C7—H7B	108.8	C16—C17—H17	108.2
C8—C7—H7B	108.8	C17—C18—H18A	109.5
H7A—C7—H7B	107.7	C17—C18—H18B	109.5
C10—C8—C7	112.56 (10)	H18A—C18—H18B	109.5
C10—C8—C9	111.31 (12)	C17—C18—H18C	109.5
C7—C8—C9	107.08 (11)	H18A—C18—H18C	109.5
C10—C8—H8	108.6	H18B—C18—H18C	109.5
C7—C8—H8	108.6	C17—C19—H19A	109.5
C9—C8—H8	108.6	C17—C19—H19B	109.5
C8—C9—H9A	109.5	H19A—C19—H19B	109.5
C8—C9—H9B	109.5	C17—C19—H19C	109.5
H9A—C9—H9B	109.5	H19A—C19—H19C	109.5
C8—C9—H9C	109.5	H19B—C19—H19C	109.5
H9A—C9—H9C	109.5	N3—C20—C21	113.98 (10)
H9B—C9—H9C	109.5	N3—C20—H20A	108.8
C8—C10—H10A	109.5	C21—C20—H20A	108.8
C8—C10—H10B	109.5	N3—C20—H20B	108.8
H10A—C10—H10B	109.5	C21—C20—H20B	108.8
C8—C10—H10C	109.5	H20A—C20—H20B	107.7
H10A—C10—H10C	109.5	C23—C21—C22	111.16 (11)
H10B—C10—H10C	109.5	C23—C21—C20	111.30 (11)
N2—C11—C12	113.34 (9)	C22—C21—C20	107.93 (10)
N2—C11—H11A	108.9	C23—C21—H21	108.8
C12—C11—H11A	108.9	C22—C21—H21	108.8
N2—C11—H11B	108.9	C20—C21—H21	108.8
C12—C11—H11B	108.9	C21—C22—H22A	109.5
H11A—C11—H11B	107.7	C21—C22—H22B	109.5
C13—C12—C14	111.00 (12)	H22A—C22—H22B	109.5
C13—C12—C11	112.09 (11)	C21—C22—H22C	109.5
C14—C12—C11	108.62 (10)	H22A—C22—H22C	109.5
C13—C12—H12	108.3	H22B—C22—H22C	109.5
C14—C12—H12	108.3	C21—C23—H23A	109.5
C11—C12—H12	108.3	C21—C23—H23B	109.5
C12—C13—H13A	109.5	H23A—C23—H23B	109.5
C12—C13—H13B	109.5	C21—C23—H23C	109.5
H13A—C13—H13B	109.5	H23A—C23—H23C	109.5
C12—C13—H13C	109.5	H23B—C23—H23C	109.5
H13A—C13—H13C	109.5	C5—N1—C1	117.21 (10)

### Hydrogen-bond geometry (Å, °)

**Table:**

*D*—H···*A*	*D*—H	H···*A*	*D*···*A*	*D*—H···*A*
C7—H7A···O2^i^	0.97	2.57	3.5226 (15)	166
C11—H11A···O1^ii^	0.97	2.55	3.4790 (15)	160

Symmetry codes: (i) *x*−1, *y*, *z*; (ii) *x*, −*y*+1/2, *z*+1/2.
